# Impact of gene variants on sex-specific regulation of human Scavenger receptor class B type 1 (SR-BI) expression in liver and association with lipid levels in a population-based study

**DOI:** 10.1186/1471-2350-11-9

**Published:** 2010-01-19

**Authors:** Ornit Chiba-Falek, Marshall Nichols, Sunil Suchindran, John Guyton, Geoffrey S Ginsburg, Elizabeth Barrett-Connor, Jeanette J McCarthy

**Affiliations:** 1 Institute for Genome Sciences and Policy, Duke University Medical Center, Durham, NC 27710, USA; 2 Division of Endocrinology, Duke University Medical Center, Durham, NC 27710, USA; 3 Department of Family and Preventive Medicine, University of California at San Diego, San Diego, CA 92121, USA

## Abstract

**Background:**

Several studies have noted that genetic variants of *SCARB1*, a lipoprotein receptor involved in reverse cholesterol transport, are associated with serum lipid levels in a sex-dependent fashion. However, the mechanism underlying this gene by sex interaction has not been explored.

**Methods:**

We utilized both epidemiological and molecular methods to study how estrogen and gene variants interact to influence *SCARB1 *expression and lipid levels. Interaction between 35 *SCARB1 *haplotype-tagged polymorphisms and endogenous estradiol levels was assessed in 498 postmenopausal Caucasian women from the population-based Rancho Bernardo Study. We further examined associated variants with overall and *SCARB1 *splice variant (SR-BI and SR-BII) expression in 91 human liver tissues using quantitative real-time PCR.

**Results:**

Several variants on a haplotype block spanning intron 11 to intron 12 of *SCARB1 *showed significant gene by estradiol interaction affecting serum lipid levels, the strongest for rs838895 with HDL-cholesterol (p = 9.2 × 10^-4^) and triglycerides (p = 1.3 × 10^-3^) and the triglyceride:HDL cholesterol ratio (p = 2.7 × 10^-4^). These same variants were associated with expression of the SR-BI isoform in a sex-specific fashion, with the strongest association found among liver tissue from 52 young women <45 years old (p = 0.002).

**Conclusions:**

Estrogen and *SCARB1 *genotype may act synergistically to regulate expression of *SCARB1 *isoforms and impact serum levels of HDL cholesterol and triglycerides. This work highlights the importance of considering sex-dependent effects of gene variants on serum lipid levels.

## Background

The scavenger receptor class B type 1 (SR-BI) is a plasma membrane protein that binds high density lipoprotein (HDL) with high affinity and mediates selective uptake of cholesterol esters by the liver[1,2]. Besides its role as a functional HDL receptor involved in reverse cholesterol transport, SR-BI also participates in the metabolism of Apolipoprotein B-containing lipoproteins, including low density lipoprotein (LDL)[3,4] and very low density lipoprotein (VLDL)[5,6]. In addition, studies have implicated SR-BI as a key co-receptor mediating infection with the hepatitis C virus[7], where chronic infection is characterized by marked lipid changes reflecting viral dependence on host lipid metabolism for replication and assembly[8]. The full length gene encoding SR-BI (gene symbol *SCARB1*) is comprised of 13 exons that are alternatively spliced to produce two major transcripts: the full length SR-BI and the splice variant SR-BII, in which exon 12 is skipped. SR-BI and SR-BII splice forms are conserved in both the mouse and rat genomes, have different tissue distributions and may influence cellular cholesterol trafficking and homeostasis in different ways[9]. SR-BII is reported to be a minor splice variant in human liver and has been shown to be less efficient at reverse cholesterol transport[10].

In humans, with the exception of genome-wide association studies, targeted investigations into the association between *SCARB1 *single nucleotide polymorphisms (SNPs) and serum lipids have been limited to a handful of polymorphisms in either exon 1, intron 5 or exon 8. These studies have found polymorphisms of *SCARB1 *associated with serum levels of triglycerides [11-14], HDL cholesterol [11-18], LDL cholesterol[11,12,17] and VLDL[12]. Interestingly, many of these studies show different effects of the polymorphisms in men and women, suggesting a possible mediating role of sex hormones[11-15,17,18].

Estrogen is known to have a profound impact on serum lipid levels, resulting in a decrease in LDL cholesterol and triglycerides, affording younger women relative protection from coronary artery disease[19]. Estrogen is also a potent regulator of *SCARB1*, influencing the relative expression of SR-BI and SR-BII isoforms[20]. In rodent models, treatment with high doses of estrogen suppressed liver expression of full length SR-BI isoform, but increased expression of the splice variant SR-BII[21,26]. Similarly, treatment of human HepG2 liver cell lines with estradiol has been shown to result in a down-regulation of SR-BI and up-regulation of the SR-BII splice form[24,27].

We previously described how the association between *SCARB1 *gene variants and HDL cholesterol levels varied by estrogen therapy status among postmenopausal women from the community-based Rancho Bernardo Study [28]. In the current study, we took a comprehensive SNP tagging approach to identify *SCARB1 *genetic variation influencing lipid levels in an estrogen dependent manner. We examined interaction between *SCARB1 *genotypes and endogenous estradiol levels among postmenopausal women, where levels of estradiol are lower than premenopausal women but not subject to monthly fluctuations. We further explored the functional consequences of *SCARB1 *genetic variation on alternative splicing of *SCARB1 *in a large series of human liver tissues. We report here how both endogenous estrogen and *SCARB1 *genotype synergistically influence serum levels of HDL cholesterol and triglycerides in human populations and how this effect may be mediated through expression of the SR-BI isoform.

## Methods

### Rancho Bernardo Study

The Rancho Bernardo Study is a population-based cohort of white men and women, established during 1972-1974 in the suburban community of Rancho Bernardo, California[29]. The present study utilizes medical history, current medication use and health behaviors assessed from standardized questionnaires, anthropometric measures, and laboratory measures collected on women between 1984 and 1987 during a follow-up visit focusing on diabetes and cardiovascular disease (visit 4). At the time of this visit, the average age of subjects was 70 years and the vast majority of women were postmenopausal. Therefore, we restricted our analyses to postmenopausal women. Subjects who participated in this visit provided morning blood samples obtained after an overnight fast and used to measure lipid and lipoprotein levels. Fasting serum cholesterol, triglyceride and HDL cholesterol levels were measured in a Center for Disease Control-Certified Lipid Research Clinic laboratory. Total cholesterol and triglyceride levels were measured by enzymatic techniques using an ABA-200 biochromatic analyzer (Abbott Laboratories, Irving, TX). HDL cholesterol was measured after precipitation of the other lipoproteins with heparin and manganese chloride. LDL cholesterol was estimated using the Friedewald formula[30]. Current use of estrogen therapy was validated by examination of pills or prescriptions brought to the clinic for that purpose and was classified as current versus non-current use. Endogenous estradiol in non-hormone using women was measured between 1992 and 1993 by radioimmunoassay after solvent extraction and column-chromatography[31] using first-thawed fasting morning specimens from the 1984-1987 venipuncture. Measurements were performed in the endocrinology research laboratory of S. S. C. Yen (Department of Reproductive Medicine, University of California-San Diego, La Jolla, CA). Serum estradiol is stable for >10 years when kept frozen at -70C (personal communication from S. Yen). The sensitivity and the intra- and inter-assay coefficients of variation, respectively, were 11 pmol/L, 5.9% and 7.1%. Because of a skewed distribution, estradiol measures were grouped into tertiles with the following cutoffs: low (7-17 pmol/L); medium (18-24 pmol/L); high (25-58 pmol/L). The 52 women with estradiol levels below the lower limit of detection (representing about 10% of the cohort) were included in the low estradiol group. Five women with estradiol levels above normal for postmenopausal women (>217 pmol/L) were excluded.

There were a total of 899 post-menopausal women who participated in visit 4 and provided a blood sample for DNA. There were 83 *SCARB1 *SNPs with minor allele frequency >0.05 in Caucasians in the HapMap database. The Tagger program in Haploview software version 3.2 [32] was used to select 35 haplotype tagged SNPs in the *SCARB1 *gene having minor allele frequencies of >0.05 and capturing all alleles with correlations (r^2^) >0.80 among Caucasians (Table 1). Of the 899 women with blood samples available, 793 were successfully genotyped for *SCARB1 *SNPs. We further excluded six women missing primary lipid data; an additional nine women on lipid lowering therapy; and 280 women currently taking estrogen therapy on whom estradiol measures were not measured, leaving a final sample of 498 women.

**Table 1:** *SCARB1 *SNPs evaluated in the Rancho Bernardo Study.

	Location	rs number	Minor allele	Allele frequency	Hardy-Weinberg p value
Tag SNPs					
	3'	rs838881	G	0.31	0.07
	3'	rs838883	T	0.07	0.26
	IVS12	rs701106	A	0.16	0.41
	IVS12	rs7977729	C	0.29	0.60
	IVS11	rs838893	T	0.31	0.41
	IVS9	rs9919713	T	0.04	0. 40
	IVS9	rs1031605	T	0.20	0.12
	IVS7	rs989892*	G	0.47	0.46
	IVS7	rs838905	C	0.04	0.31
	IVS6	rs838900	G	0.08	0.90
	IVS2	rs4765615	T	0.47	0.40
	IVS2	rs745529	T	0.37	0.006
	IVS1	rs11057830	A	0.15	0.45
	IVS1	rs10846745	G	0.44	0.85
	IVS1	rs7135223	A	0.40	0.12
	IVS1	rs12581963	A	0.08	0.69
	IVS1	rs12580803	G	0.17	0.59
	IVS1	rs10773107	A	0.48	0.50
	IVS1	rs10744182	C	0.42	0.84
	IVS1	rs4765180	T	0.43	0.97
	IVS1	rs7954519	G	0.20	0.44
	IVS1	rs4765621	T	0.33	0.12
	IVS1	rs12229555	C	0.21	0.86
	IVS1	rs10773109	G	0.45	0.90
	IVS1	rs12582221	A	0.44	0.48
	IVS1	rs12370382	T	0.39	0.84
	IVS1	rs3924313	A	0.33	0.51
	IVS1	rs11057851	A	0.11	0.53
	IVS1	rs11057852	T	0.05	0.76
	IVS1	rs10773111	T	0.42	0.38
	IVS1	rs4765181	A	0.39	0.99
	IVS1	rs11615630	T	0.40	1.00
	5'	rs4379922	C	0.37	1.00
	5'	rs10846760	T	0.36	1.00
Additional SNPs					
	IVS11	rs838892	A	0.31	0.47
	IVS11	rs838895	G	0.32	0.82
	IVS11	rs838896	C	0.34	0.52

*Proxy SNP for exon 8 SNP evaluated in previous studies (rs5888)

### Liver Samples

Post-mortum liver tissues from 93 otherwise healthy Caucasian adults were purchased from the National Institute of Child Health and Human Development Brain and Tissue Bank for Developmental Disorders (University of Maryland, Baltimore, MD). All had a post mortem interval (PMI) <24 hours. A demographic description is included in Table 2. The human hepatoma (HepG2) cell line was obtained from American Type Culture Collection (Manassas, VA).

**Table 2:** Demographic characteristics and *SCARB1 *SNP minor allele carrier frequencies in 93 post-mortem liver samples, obtained from otherwise healthy Caucasian adults.

Characteristic	Males (all) (n = 41)	Females (all) (n = 52)	Females < 45 years old (n = 43)
Age (years)	40.9 ± 12.1	32.2 ± 13.8	27.8 ± 9.4
PMI (hours)	14.1 ± 5.9	17.2 ± 7.3	17.0 ± 7.5
rs7977729 (C)	0.41	0.50	0.49
rs838891 (G)	0.56	0.54	0.53
rs838892 (A)	0.56	0.53	0.52
rs838893 (T)	0.56	0.52	0.51
rs838895 (G)	0.54	0.54	0.53
rs838896 (C)	0.51	0.58	0.58
rs10846744 (G)	0.39	0.25	0.28

PMI = post mortem interval. Age and PMI are presented as means ± standard deviation.

### RNA extraction and cDNA synthesis from liver tissue

Total RNA was extracted from liver sample (100 mg) using TRIzol reagent (Invitrogen, Carlsbad, CA) followed by purification with RNeasy kit using an on-column DNase treatment (Qiagen, Inc., Valencia, CA) following the manufacturer's protocol. RNA concentration was determined spectrophotometrically at 260 nm, while the quality of the purification was determined by 260 nm/280 nm ratio. Additionally, quality of sample and lack of significant degradation products was confirmed on an Agilent Bioanalyzer. Next, cDNA was synthesized using MultiScribe RT enzyme (Applied Biosystems, Foster City, CA) under the following conditions: 10 min at 25°C and 120 min at 37°C.

### Real time PCR

Real-time PCR was used to quantify human *SCARB1 *mRNA levels (SR-BI isoform, SR-BII isoform and overall *SCARB1*) using commercially available TaqMan assays. Briefly, each liver sample was assayed by quantitative real-time PCR using the ABI 7900 to determine the level of the target message relative to mRNA encoding the human peptidylprolyl isomerase A *(PPIA)*. This housekeeping gene has been shown to be among the most stable in studies of mouse liver, making it ideal for normalization in liver gene expression studies[33]. Each cDNA (50 ng) was amplified in duplicate using TaqMan Gene Expression PCR master mix reagent (Applied Biosystems, Foster City, CA) under the following conditions: 2 min at 50°C, 10 min at 95°C, 40 cycles: 15 sec at 95°C, and 1 min at 60°C. The different target cDNAs were amplified using the following ABI MGB probe and primer set assays (Applied Biosystems, Foster City, CA): ID HS00969822_m1 for overall *SCARB1*, targeting the junction of *SCARB1 *exon 2-3 which is conserved across all isoforms; HS00969819_m1 for SR-BI (includes exon 12), targeting the junction of exons 11-12; and a custom assay designed for SR-BII (excludes exon 12), with forward primer 5'TCCCTGTCATCTGCCAAATCC-3', reverse primer 5'-GGCTGGCTCACGGTGT-3' and probe 5'-CCTCAGGACCTTGGCTCC-3' targeting the junction of exons 11-13. All three mRNA assays were normalized to a *PPIA *mRNA control (ABI MGB probe and primer set assay ID Hs99999904_m1).

The data were analyzed using the ΔΔCt method, with a threshold set in the linear range of amplification. The cycle number at which any particular sample crossed that threshold (Ct) was then used to determine fold difference. Fold difference was calculated as 2^-ΔΔCt^; ΔCt = [Ct(*target*)-Ct (*PPIA*)]. ΔΔCt = [ΔCt(sample)]-[ΔCt(calibrator)], where target is *SCARB1*, SR-BI or SR-BII. The calibrator was a single, randomly selected control liver sample used in each plate for normalization within and across runs and used in creation of standard curves. Each sample was run in duplicate in two independent plates, over all 4 repeats. The ΔΔCt results obtained with the four repeats were average to determine the fold expression level used in the association analysis.

We controlled for DNA contamination by running three randomly selected, RNA control samples that were not converted to cDNA and no-cDNA/RNA sample in each plate. No observable amplification was detected. In addition for assay validation we generated standard curves for each target and reference assay, using different amounts of human liver total RNA (1-1000 ng). In addition, the slope of the relative efficiency plot for each target and internal control were determined to validate the assays, i.e. *SCARB1*, SR-BI or SR-BII and *PPIA*. The slope in the relative efficiency plot for each target and *PPIA *were determined, and showed a standard value (<0.1) required for the validation of the relative quantitative method. (Additional file 1, Figure S1).

### SCARB1 genotyping

Genomic DNA was isolated using conventional protocol by Qiagen kits (Qiagen, Inc., Valencia, CA). *SCARB1 *genotypes were assayed in genomic DNA isolated from frozen whole blood samples from subjects in the Rancho Bernardo Study and from the liver tissues and HepG2 cell line using either the Sequenom iPLEX™ multiplex mass spectrometry genotyping system (Sequenom, Inc, La Jolla, CA) or using the 5' nuclease assay with allele specific TaqMan probes[34]. The percent missing genotypes and Hardy-Weinberg Equilibrium were evaluated using Haploview software[32] as a means of quality control.

### Statistical analysis

SAS statistical software, Version 9.1 (SAS Institutes, Cary, NC) was used for all statistical analyses. The ggplot2 package[35] of the R language was also used for plots[36]. The association between each *SCARB1 *SNP and levels of total cholesterol, LDL cholesterol, triglycerides, HDL cholesterol and the triglyceride:HDL cholesterol ratio in the Rancho Bernardo Study was assessed with Analysis of Variance (ANOVA). Triglycerides, HDL cholesterol, LDL cholesterol, and the TG/HDL cholesterol ratio were natural log (ln) transformed to improve normality of the residuals. SNP genotypes were coded in an additive model, except for SNPs where the minor allele frequency was below 5%, in which case heterozygous and homozygous variant genotypes were pooled to test a dominant model. All models controlled for age (continuous) and current estrogen therapy status. Interactions between SNPs and estradiol levels (based on tertile cutoffs) were determined by introduction of a cross-product term in the model.

Differences in overall *SCARB1*, SR-BI and SR-BII expression in liver samples were examined by sex and by presence/absence of *SCARB1 *allelic variants using standard linear regression analysis, controlling for post-mortum interval and age. Genotypes were coded in a dominant model (homozygous variant and heterozygote genotypes pooled) because of small numbers. Genotype by sex interaction was determined by inclusion of a cross product term in the model. Expression levels were natural log (ln) transformed for analysis. The proportion of SR-BI and SR-BII out of total *SCARB1 *was determined by calculating the ΔΔct ratios (i.e. SR-BI or SR-BII/overall *SCARB1)*.

This study was approved by the Institutional Review Boards at Duke University.

## Results

### Genotype associations with lipid traits in women from the Rancho Bernardo Study

**Figure 1:**
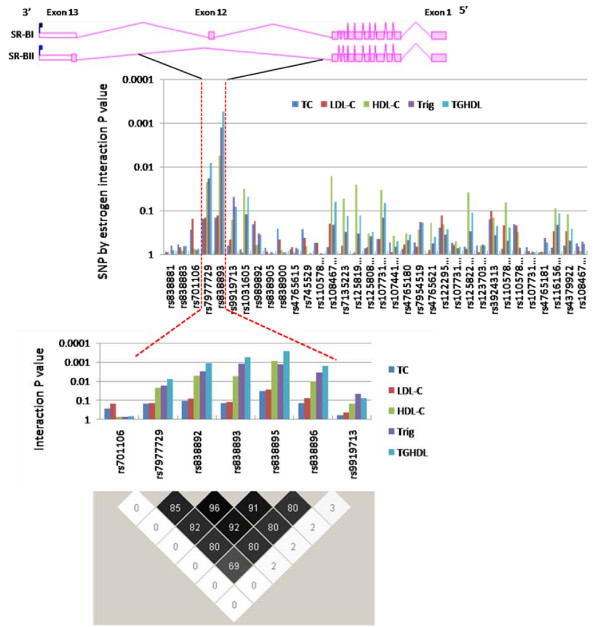
***SCARB1 *SNPs associated with lipid levels in an estradiol-dependent manner in postmenopausal women**. Top panel - Intron/exon structure of *SCARB1 *isoforms, SR-BI and SR-BII; Middle two panels - P value for interaction between *SCARB1 *SNPs and endogenous estradiol levels for serum lipid levels in the Rancho Bernardo Study; Bottom panel - haplotype block representing associated interval (numbers are r^2 ^values measuring pairwise linkage disequilibrium between SNPs). SNPs are ordered based on their position in the gene and from left (3') to right (5') ends of the gene. TC = total cholesterol; LDL-C = low density lipoprotein cholesterol; HDL-C = high density lipoprotein cholesterol; Trig = triglycerides; TGHDL = triglyceride:HDL-C ratio.

**Figure 2:**
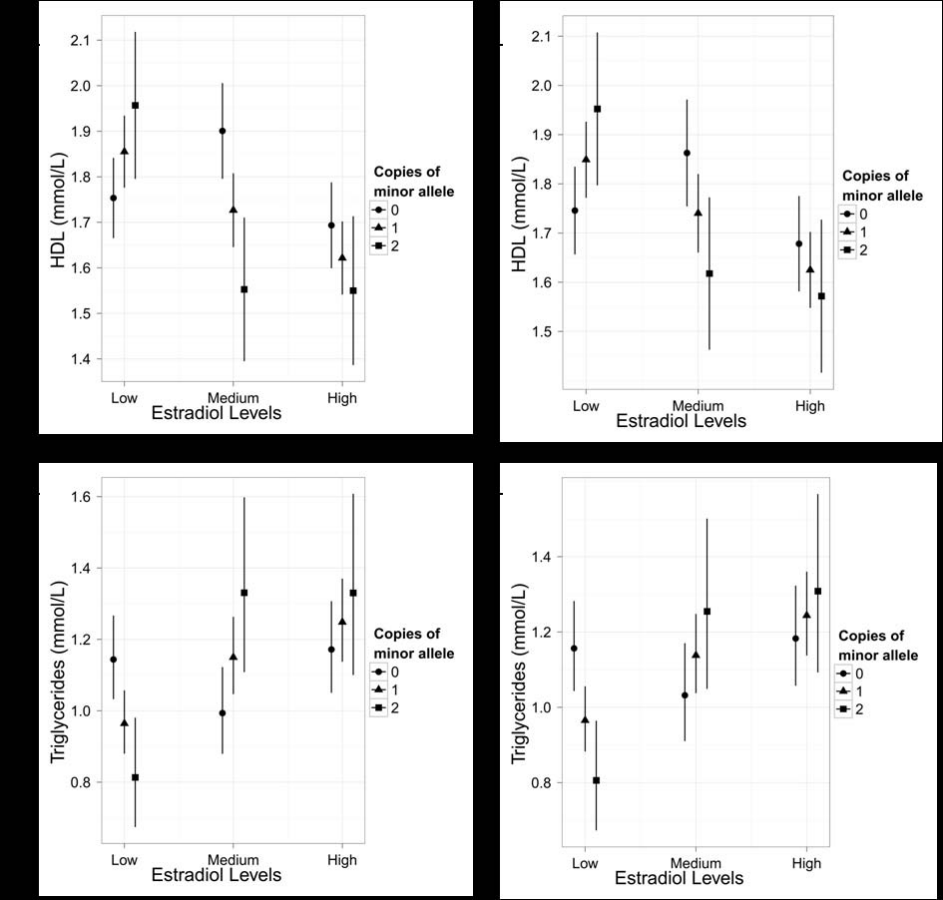
**Effect of *SCARB1 *genotype on serum lipid levels by tertile of endogenous estradiol**. Shown are mean values of HDL cholesterol and triglycerides with 95% confidence intervals for subjects with 0, 1 or 2 copies of the rs838895 (panels A and B) and rs838896 (panels C and D) minor alleles. Endogenous estradiol tertiles were: low (7-17 pmol/L); medium (18-24 pmol/L); high (25-58 pmol/L).

Minor allele frequencies for the *SCARB1 *SNPs typed in this study ranged from 0.04 to 0.44. The average call rate was 97% and one SNP had nominally significant (p < 0.006) deviation from Hardy-Weinberg equilibrium (Table 1). Characteristics of women from the Rancho Bernardo Study are shown in Table 3. We modeled the interaction between the *SCARB1 *SNPs and estradiol levels on serum total cholesterol, triglycerides, HDL cholesterol, LDL cholesterol and the triglyceride:HDL cholesterol ratio among women not currently taking estrogen therapy. Two SNPs on the same haplotype block showed significant evidence of genotype by estradiol interaction (p < 0.05). The tag SNP rs838893 showed the strongest evidence of interaction for HDL cholesterol (p = 0.006), triglycerides (p = 0.001) and the triglyceride:HDL cholesterol ratio (p = 0.0005). This common variant (minor allele frequency 0.31) lies on a haplotype block spanning intron 11 to intron 12 of *SCARB1*. We examined additional SNPs on this block, all of which showed a similar pattern of significant associations (Figure 1). Figure 2 illustrates how at low levels of estradiol, the variant allele, G', of the most significant of these SNPs, rs838895, was associated with lower levels of triglycerides and high levels of HDL cholesterol, but at increasing levels of estradiol, the opposite was found. Importantly, this SNP showed no association with estradiol levels (p = 0.40). Results for another significant SNP, rs838896, are also shown in Figure 2 for comparison with results from the livers, presented below. Similar trends were seen for all assayed SNPs on that haplotype block. No significant interaction was found between any of the *SCARB1 *tagging SNPs and current use of estrogen therapy for HDL-C (p = 0.34) or triglycerides (p = 0.53).

**Table 3:** Baseline characteristics of postmenopausal Caucasian women from the Rancho Bernardo Study in the overall cohort and by low, moderate or high estradiol (E2) tertile in the subset with estradiol measures available.

Characteristic (mean ± SD)	All women	Low E2	Mod E2	High E2
Sample size	(784)	(180)	(140)	(173)
Age (years)	69.1 ± 8.6	71.1 ± 8.2	70.8 ± 8.4	68.5 ± 9.2
Waist circumference (cm)	78.0 ± 9.1	75.8 ± 8.0	78.2 ± 8.6	80.9 ± 9.6
BMI (kg/m^2^)	24.1 ± 3.6	23.0 ± 3.0	23.9 ± 2.9	25.5 ± 4.0
Total cholesterol (mmol/L)	5.9 ± 1.0	5.9 ± 1.1	6.1 ± 1.0	6.0 ± 0.94
HDL cholesterol (mmol/L)	1.8 ± 0.5	1.8 ± 0.45	1.8 ± 0.47	1.6 ± 0.43
LDL cholesterol (mmol/L)	3.5 ± 1.0	3.6 ± 1.0	3.7 ± 1.0	3.8 ± 0.9
Triglycerides (mmol/L)*	1.1 ± 0.8	1.0 ± 0.7	1.1 ± 0.8	1.2 ± 1.0
Estradiol levels (pmol/L)*	-	11.0 ± 7.3	18.4 ± 3.7	29.4 ± 11

* Median and interquartile range

### Expression levels and presence of alternative splice variants in liver tissue

Given the existence of alternative splice forms of *SCARB1 *involving inclusion/exclusion of exon 12, we hypothesized that a SNP located within the associated haplotype block may modulate splicing efficiency. We quantified the amounts of the full-length SR-BI isoform (including exon 12), the alternative SR-BII isoform (exon 12 skipping) and overall *SCARB1 *mRNA (all splice forms) in human liver tissues from 93 healthy individuals. SR-BI showed higher expression levels than SR-BII, consistent with other studies, but accounted for less than half of the overall *SCARB1 *expression: on average SR-BI accounted for 39 ± 19% and SR-BII 3.5 ± 1.8% of overall *SCARB1 *expression.

### The effect of sex and genotype on alternative splicing of SCARB1 in human liver tissues

In order to determine the functional consequences of *SCARB1 *SNPs, we carried out genetic association analysis of the associated SNPs and expression of *SCARB1*. We genotyped seven SNPs, including six within the associated haplotype block, which spans intron 11 to intron 12 of *SCARB1*, and one outside the region in 93 commercially available liver samples. All SNPs were in Hardy-Weinberg Equilibrium and showed linkage disequilibrium patterns consistent with those seen in the Rancho Bernardo Study (not shown). One outlier sample was removed after performing linear regression diagnostics and the reference liver sample was also not included, leaving a sample size of 91 (57% female). Because of the apparent estrogen dependency of *SCARB1 *variants on lipid levels, we examined primary sex effects, primary genotype effects and interactive effects between sex and *SCARB1 *genotype on mRNA expression.

**Figure 3:**
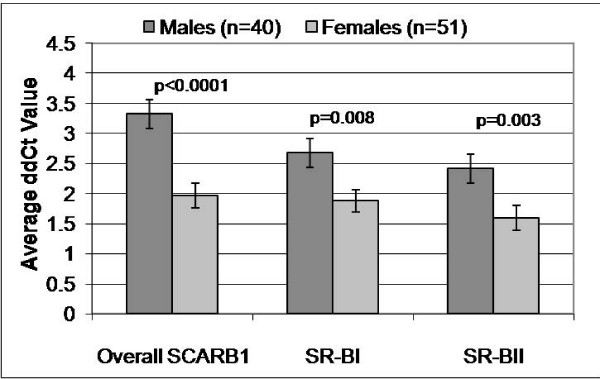
**Sex differences in mRNA expression in human liver tissue**. Shown are average ΔΔCt values with standard error bars for full length SR-BI isoform, SR-BII and overall *SCARB1*, all relative to PPIA control in 91 human liver tissue, controlling for effects of age and post-mortem interval.

**Figure 4:**
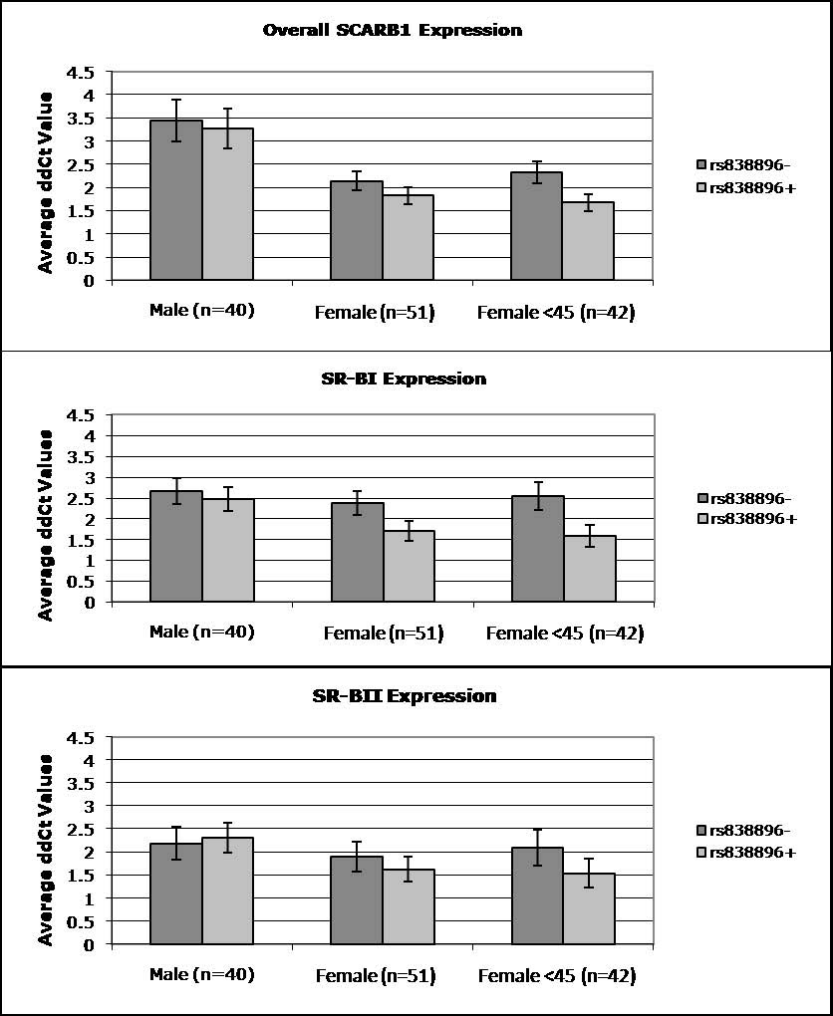
**mRNA expression in human liver tissue by *SCARB1 *genotype and sex**. Results shown are average ΔΔCt values with standard error bars for A.) Full length SR-BI isoform; B.) SR-BII and C.) overall *SCARB1*, all relative to PPIA control. Rs838896+ refers to carrier of the G allele (GG and GC); Rs838896- refers to genotype CC.

Previous studies found that estrogen treatment of human HepG2 cells results in a decrease in SR-BI and increase of SR-BII[24,27]. First, as a proxy for estrogen effects, we examined male-female differences in liver expression of SR-BI, SR-BII and overall *SCARB1*. We found significantly *lower *levels of overall *SCARB1*, SR-BI isoform and SR-BII isoform in female versus male livers (Figure 3). Next, we examined the effect of each of the seven SNPs on expresssion in the combined liver samples from men and women, but no significant associations were found. We then repeated this analysis, assessing SNP by sex interaction for all SNPs. We found modest evidence of interaction between sex and *SCARB1 *SNP rs838896 influencing expression of the SR-BI isoform (p = 0.09), which became stronger when restricting analysis to subjects ≤45 years old (p = 0.04), leading us to carry out sex-stratified analyses. As shown in Figure 4, presence (+) of the rs838896 minor allele, C, was associated with significantly lower levels of SR-BI in liver tissue from women (p = 0.01) but not in men (p = 0.65). Restricting the analysis to liver tissue from women <45 years of age, the genotype association became stronger: in younger women, presence of at least one copy of the rs838896C allele conferred a 38% lower level of SR-BI (p = 0.002), a non-signfiicant 26% lower level of SR-BII (p = 0.16) and a 28% lower level of overall *SCARB1*(p = 0.02), compared to young women not carrying this allele. Similar, but less significant associations were seen for other SNPs on the haplotype block (rs7977729, rs838891, rs838892, rs838893, rs838895, and rs838896). It is noteworthy that rs838895, which showed the strongest association with lipids in the Rancho Bernardo Study, did not reach significance in the liver gene expression analysis (e.g. in females <45, the p values were 0.07, 0.13 and 0.54 for association with overall *SCARB1*, SR-BI and SR-BII expression). This may be due to differences in linkage disequilibrium between rs838895 and rs838896 in the two studies: r^2 ^= 0.80 in Rancho Bernardo and r^2 ^= 0.62 in the livers. No associations were found for the one SNP outside of the block (rs10846744). We then compared relative levels of each splice form (ratio to overall *SCARB1 *expression) in young women and found no significant differences by genotype: Carriers of the rs838896 minor allele (rs838896+) had on average 41 ± 4% SR-BI and 3.9 ± 0.4 % SR-BII while non-carriers (rs838896-) had average levels of 47 ± 5% and 3.6 ± 0.4%, respectively.

## Discussion

The results of our study in a community-based cohort of post-menopausal women demonstrate that polymorphisms of *SCARB1 *are associated with HDL cholesterol and triglyceride levels in an endogenous estrogen-dependent fashion. Specifically, variant alleles of *SCARB1 *in a block encompassing intron 11 to intron 12 were associated with increased triglycerides and decreased HDL cholesterol in the presence of high levels of endogenous estradiol, but the opposite was seen at lower levels of estradiol. Importantly, these effects were observed among post-menopausal women who already have low levels of estradiol, suggesting that small changes in endogenous estradiol can impact the association of *SCARB1 *genotype on serum lipid levels.

In contrast, we did not find evidence of interaction between these same genotypes and current use of oral estrogen therapy, as one might expect, nor did we find evidence of genotype by endogenous estradiol interaction for two SNPs reported to interact with oral estrogen therapy in our previous study of HDL cholesterol (data not shown). These discrepancies may be related to the marked perturbation of hepatic metabolism produced by oral estrogen therapy, including increased plasma levels of VLDL, and HDL, and reduced plasma LDL[37,38]. Oral estrogen is administered in high doses due to poor systemic bioavailability averaging 5%[39] and thus hepatocyte exposure to oral estrogen may be magnified, exceeding the physiological range. Another difference between oral and endogenous estrogen is that the former has typically involved equine estrogens (particularly during the period of sample collection for this study) whereas estradiol is the most active endogenous estrogen. Their effects on gene expression relevant to lipid metabolism may be quite different. Another possibility is that the SNPs that interacted with estrogen therapy in our previous study could reflect other differences between users and non-users of estrogen therapy, the former being younger and having lower fasting glucose[28], as well as having lower testosterone (unpublished data).

We further demonstrate in the current study that in liver tissues from young, adult females (but not males) the *SCARB1 *rs838896 minor allele from the identified haplotype block is associated with lower overall expression, driven in large part by the SR-BI isoform. Hence, increased expression of SR-BI may underlie the protective lipid profiles observed in women with both high levels of estradiol and homozygous for the rs838896 wildtype allele as observed in the Rancho Bernardo Study, and could account for underlying sex differences in *SCARB1 *genetic associations with lipids described in previous studies[11-15,17,18].

Previous studies in rodent models and human HepG2 cell lines indicate that estrogen down-regulates SR-BI and up-regulates SR-BII isoforms[24,27]. Thus, one would expect that women (specifically pre-menopausal women with higher levels of estrogen) would have lower levels of SR-BI and higher levels of SR-BII than men. Our findings in a series of human liver tissues confirm that females have significantly lower levels of hepatic SR-BI expression compared to males, but SR-BII levels were not higher; in fact levels of SR-BII were lower in females, especially among carriers of the *SCARB1 *rs838896 minor allele (the same genotype of HepG2 cell lines, data not shown). Although experimental results in human liver tissue may not equate with those in rodent liver or cultured human HepG2 cell lines, these discrepant findings warrant further investigation. In the study by Graf[27], antibodies specific to the 3' end of the *SCARB1 *protein were used to distinguish SR-BI from SR-BII in human HepG2 cell lines treated with 17 estradiol. In the study by Zhang[24], treatment of HepG2 cell lines under the same conditions as Graf *et al*. did not produce any changes in SR-BI or SR-BII. Only after subsequent introduction of estrogen receptor and treatment with synthetic equine estrogen did they recapitulate the increase in SR-BII expression noted by Graf *et al*. However, SR-BII was not measured directly, but was determined by calculating the difference between overall *SCARBI *and SR-BI levels. SR-BII, resulting from skipping of exon 12, is the most studied alternatively splice form, but may not be the only splice form. Additional splice forms have been described in genomic databases such as Entrez Gene Aceview[40], including one form that differs dramatically in the 3' end of the gene, missing both exons 12 and 13 and retaining a large segment of intron 11 as coding. We confirmed the expression of this splice form in human brain and liver tissues (data not shown). Thus, differences in our findings from others may relate to the method of detection of SR-BII expression and the possible existence of additional isoforms of *SCARB1 *not accounted for in any published analyses to date.

Recently, Zhang and colleagues proposed that estrogen regulation of alternative splicing of *SCARB1 *in the rat occurs via regulatory splicing factors that interact with regulatory sequences in intron 11 of *SCARB1*[24,41]. The human *SCARB1 *polymorphisms most strongly associated with estrogen-dependent splicing and lipid traits in our study are located on a haplotype block spanning intron 11 to intron 12. The coincidental location of the putative regulatory region identify by Zhang and the presence of SNPs controlling SR-BI isoform expression in our study lead us to suspect that the polymorphism underlying our observed association impacts the estrogen-dependent regulation of *SCARB1 *expression, perhaps of specific isoforms. Our data do not support an effect of *SCARB1 *SNPs on relative amounts of SR-BI or SR-BII in liver, but the possibility that other isoforms are impacted by these gene variants should be explored. Whether the rs838896 variant is the causal variant or in linkage disequilibrium with the causal variant remains to be determined in follow up functional studies using cell culture and other systems.

Both HDL cholesterol and triglyceride levels are sexually dimorphic traits: levels of HDL cholesterol are 20-25% higher in women than men and these differences persist over time; triglyceride levels, on the other hand, start out slightly lower in women compared to men in young adulthood, but around the time of menopause, triglyceride levels rise dramatically in women[42]. We propose that this rise in triglycerides may be attributable in part to estrogen regulation of *SCARB1*, and that the protection afforded by estrogen in younger women may be limited to women with a certain *SCARB1 *genotype. It is interesting to note that *SCARB1 *is also a co-receptor for the Hepatitis C virus (HCV) and that increased expression of both SR-BI and SR-BII isoforms increase infectivity *in vitro*[43]. Additionally, women generally have better outcomes of HCV infection, perhaps related to estrogen [44], leading us to speculate on the role of estrogen-mediated expression of *SCARB1 *in outcomes of Hepatitis C infection.

Our study of the phenotypic consequences of *SCARB1 *variation was carried out in a community-based sample of post-menopausal Caucasian women who were not selected based on any disease phenotype. The samples and data were collected before the widespread use of lipid lowering therapy, thus reducing confounding by treatment. Yet, there are some caveats of our study, including that estradiol assays were measured only once. Although single measurements of most hormones reliably characterize average levels over a two to three year period, estradiol levels are less reproducible[45,46]. However, estradiol was measured in morning fasting samples, and among postmenopausal women estradiol does not show significant diurnal variation[47]. Moreover, it should be noted that this was an elderly population of women, with relatively low levels of estradiol; we might expect estrogen effects to be more pronounced in a younger population. As with most large scale SNP association studies, ours is also prone to false positive associations, the result of multiple testing. Replication of the observed associations in other study populations is warranted.

## Conclusions

In summary, our work has detected sex-specific expression of *SCARB1 *isoforms *in vivo *in human liver tissue and identified polymorphisms in intron 11 of the gene that modify this effect. We further found these same genetic variants to influence triglyceride and HDL cholesterol levels in an endogenous estrogen-dependent manner in a human population study. Thus, estrogen regulation of *SCARB1 *may contribute to the observed sexual dimorphism of lipid levels and highlights the importance of considering sex-dependent effects of gene variants on serum lipid levels.

## Competing interests

The authors declare that they have no competing interests.

## Authors' contributions

OCF contributed to the conception and design, oversaw the molecular genetic studies, and contributed to drafting of the manuscript; MN carried out the molecular genetic analysis; SS carried out the genetic association analysis; JG, GG and EBC participated in interpretation of data and manuscript revisions; JJM conceived and designed the study, drafted the manuscript and oversaw all genetic analyses. All authors have read and approved the final manuscript.

## Pre-publication history

The pre-publication history for this paper can be accessed here:

http://www.biomedcentral.com/1471-2350/11/9/prepub

## Supplementary Material

Additional file 1 **Figure S1. Validation curves of the ΔΔ real-time gene expression assays**. Shown are curves for overall *SCARB1*, SR-BI and SR-BII versus endogenous control (PPIA). Relative efficiency plots were formed by plotting the log input amount (ng of total RNA) versus the ΔCt (Ct *SCARB1 *- Ct *PPIA*), for example. The slopes are <0.1 which indicates the validation of the ΔΔCt calculation relative to the reference controls in the range between 1-100 ng RNA. Click here for file

## References

[B1] Acton S Rigotti A Landschulz KT Xu S Hobbs HH Krieger M Identification of scavenger receptor SR-BI as a high density lipoprotein receptor Science 1996 271 5248 518 520 10.1126/science.271.5248.518 8560269

[B2] Murao K Terpstra V Green SR Kondratenko N Steinberg D Quehenberger O Characterization of CLA-1, a human homologue of rodent scavenger receptor BI, as a receptor for high density lipoprotein and apoptotic thymocytes J Biol Chem 1997 272 28 17551 17557 10.1074/jbc.272.28.17551 9211901

[B3] Trigatti BL Rigotti A Braun A Cellular and physiological roles of SR-BI, a lipoprotein receptor which mediates selective lipid uptake Biochim Biophys Acta 2000 1529 1-3 276 286 11111095 10.1016/s1388-1981(00)00154-2

[B4] Brodeur MR Luangrath V Bourret G Falstrault L Brissette L Physiological importance of SR-BI in the in vivo metabolism of human HDL and LDL in male and female mice J Lipid Res 2005 46 4 687 696 10.1194/jlr.M400165-JLR200 15654132

[B5] Van Eck M Hoekstra M Out R Bos IS Kruijt JK Hildebrand RB Van Berkel TJ Scavenger receptor BI facilitates the metabolism of VLDL lipoproteins in vivo J Lipid Res 2008 49 1 136 146 10.1194/jlr.M700355-JLR200 17954936

[B6] Hu L Hoogt CC van der Espirito Santo SM Out R Kypreos KE van Vlijmen BJ Van Berkel TJ Romijn JA Havekes LM van Dijk KW The hepatic uptake of VLDL in lrp-ldlr-/-vldlr-/- mice is regulated by LPL activity and involves proteoglycans and SR-BI J Lipid Res 2008 49 7 1553 1561 10.1194/jlr.M800130-JLR200 18367731

[B7] Dreux M Dao Thi VL Fresquet J Guerin M Julia Z Verney G Durantel D Zoulim F Lavillette D Cosset FL Receptor complementation and mutagenesis reveal SR-BI as an essential HCV entry factor and functionally imply its intra- and extra-cellular domains PLoS Pathog 2009 5 2 e1000310 10.1371/journal.ppat.1000310 19229312 PMC2636890

[B8] Burlone ME Budkowska A Hepatitis C virus cell entry: role of lipoproteins and cellular receptors J Gen Virol 2009 90 Pt 5 1055 1070 10.1099/vir.0.008300-0 19264629

[B9] Eckhardt ER Cai L Sun B Webb NR Westhuyzen DR van der High density lipoprotein uptake by scavenger receptor SR-BII J Biol Chem 2004 279 14 14372 14381 10.1074/jbc.M313793200 14726519

[B10] Webb NR Connell PM Graf GA Smart EJ de Villiers WJ de Beer FC Westhuyzen DR van der SR-BII, an isoform of the scavenger receptor BI containing an alternate cytoplasmic tail, mediates lipid transfer between high density lipoprotein and cells J Biol Chem 1998 273 24 15241 15248 10.1074/jbc.273.24.15241 9614139

[B11] Acton S Osgood D Donoghue M Corella D Pocovi M Cenarro A Mozas P Keilty J Squazzo S Woolf EA Association of polymorphisms at the SR-BI gene locus with plasma lipid levels and body mass index in a white population Arterioscler Thromb Vasc Biol 1999 19 7 1734 1743 10397692 10.1161/01.atv.19.7.1734

[B12] Tai ES Adiconis X Ordovas JM Carmena-Ramon R Real J Corella D Ascaso J Carmena R Polymorphisms at the SRBI locus are associated with lipoprotein levels in subjects with heterozygous familial hypercholesterolemia Clin Genet 2003 63 1 53 58 10.1034/j.1399-0004.2003.630108.x 12519372

[B13] McCarthy JJ Lehner T Reeves C Moliterno DJ Newby LK Rogers WJ Topol EJ Association of genetic variants in the HDL receptor, SR-B1, with abnormal lipids in women with coronary artery disease J Med Genet 2003 40 6 453 458 10.1136/jmg.40.6.453 12807968 PMC1735488

[B14] McCarthy JJ Lewitzky S Reeves C Permutt A Glaser B Groop LC Lehner T Meyer JM Polymorphisms of the HDL receptor gene associated with HDL cholesterol levels in diabetic kindred from three populations Hum Hered 2003 55 4 163 170 10.1159/000073986 14566094

[B15] Hong SH Kim YR Yoon YM Min WK Chun SI Kim JQ Association between HaeIII polymorphism of scavenger receptor class B type I gene and plasma HDL-cholesterol concentration Ann Clin Biochem 2002 39 Pt 5 478 481 10.1258/000456302320314485 12227853

[B16] Hsu LA Ko YL Wu S Teng MS Peng TY Chen CF Lee YS Association between a novel 11-base pair deletion mutation in the promoter region of the scavenger receptor class B type I gene and plasma HDL cholesterol levels in Taiwanese Chinese Arterioscler Thromb Vasc Biol 2003 23 10 1869 1874 10.1161/01.ATV.0000082525.84814.A9 12816880

[B17] Morabia A Ross BM Costanza MC Cayanis E Flaherty MS Alvin GB Das K James R Yang AS Evagrafov O Population-based study of SR-BI genetic variation and lipid profile Atherosclerosis 2004 175 1 159 168 10.1016/j.atherosclerosis.2004.03.014 15186961

[B18] Roberts CG Shen H Mitchell BD Damcott CM Shuldiner AR Rodriguez A Variants in scavenger receptor class B type I gene are associated with HDL cholesterol levels in younger women Hum Hered 2007 64 2 107 113 10.1159/000101962 17476110 PMC2861530

[B19] Bush TL Fried LP Barrett-Connor E Cholesterol, lipoproteins, and coronary heart disease in women Clin Chem 1988 34 8B B60 70 3042201

[B20] Lopez D McLean MP Estrogen regulation of the scavenger receptor class B gene: Anti-atherogenic or steroidogenic, is there a priority? Mol Cell Endocrinol 2006 247 1-2 22 33 10.1016/j.mce.2005.10.005 16297529

[B21] Landschulz KT Pathak RK Rigotti A Krieger M Hobbs HH Regulation of scavenger receptor, class B, type I, a high density lipoprotein receptor, in liver and steroidogenic tissues of the rat J Clin Invest 1996 98 4 984 995 10.1172/JCI118883 8770871 PMC507514

[B22] Fluiter K Westhuijzen DR van der van Berkel TJ In vivo regulation of scavenger receptor BI and the selective uptake of high density lipoprotein cholesteryl esters in rat liver parenchymal and Kupffer cells J Biol Chem 1998 273 14 8434 8438 10.1074/jbc.273.14.8434 9525955

[B23] Stangl H Graf GA Yu L Cao G Wyne K Effect of estrogen on scavenger receptor BI expression in the rat J Endocrinol 2002 175 3 663 672 10.1677/joe.0.1750663 12475377

[B24] Zhang X Moor AN Merkler KA Liu Q McLean MP Regulation of alternative splicing of liver scavenger receptor class B gene by estrogen and the involved regulatory splicing factors Endocrinology 2007 148 11 5295 5304 10.1210/en.2007-0376 17673517

[B25] Fluiter K Sattler W De Beer MC Connell PM Westhuyzen DR van der van Berkel TJ Scavenger receptor BI mediates the selective uptake of oxidized cholesterol esters by rat liver J Biol Chem 1999 274 13 8893 8899 10.1074/jbc.274.13.8893 10085133

[B26] Serougne C Feurgard C Hajri T Champarnaud G Ferezou J Mathe D Lutton C Catabolism of HDL1 cholesteryl ester in the rat. Effect of ethinyl estradiol treatment C R Acad Sci III 1999 322 7 591 596 10488433 10.1016/s0764-4469(00)88529-7

[B27] Graf GA Roswell KL Smart EJ 17beta-Estradiol promotes the up-regulation of SR-BII in HepG2 cells and in rat livers J Lipid Res 2001 42 9 1444 1449 11518764

[B28] Richard E von Muhlen D Barrett-Connor E Alcaraz J Davis R McCarthy JJ Modification of the effects of estrogen therapy on HDL cholesterol levels by polymorphisms of the HDL-C receptor, SR-BI: the Rancho Bernardo Study Atherosclerosis 2005 180 2 255 262 10.1016/j.atherosclerosis.2004.12.013 15910850

[B29] Criqui MH Barrett-Connor E Austin M Differences between respondents and non-respondents in a population-based cardiovascular disease study Am J Epidemiol 1978 108 5 367 372 727205 10.1093/oxfordjournals.aje.a112633

[B30] Friedewald WT Levy RI Fredrickson DS Estimation of the concentration of low-density lipoprotein cholesterol in plasma, without use of the preparative ultracentrifuge Clin Chem 1972 18 6 499 502 4337382

[B31] Anderson DC Hopper BR Lasley BL Yen SS A simple method for the assay of eight steroids in small volumes of plasma Steroids 1976 28 2 179 196 10.1016/0039-128X(76)90108-2 973234

[B32] Barrett JC Fry B Maller J Daly MJ Haploview: analysis and visualization of LD and haplotype maps Bioinformatics 2005 21 2 263 265 10.1093/bioinformatics/bth457 15297300

[B33] Tatsumi K Ohashi K Taminishi S Okano T Yoshioka A Shima M Reference gene selection for real-time RT-PCR in regenerating mouse livers Biochem Biophys Res Commun 2008 374 1 106 110 10.1016/j.bbrc.2008.06.103 18602371

[B34] Livak KJ Allelic discrimination using fluorogenic probes and the 5' nuclease assay Genet Anal 1999 14 5-6 143 149 10084106 10.1016/s1050-3862(98)00019-9

[B35] Wickham H R package ggplot2: An implementation of the Grammar of Graphicsversion 0.8.1. edn 2008

[B36] Team RDC R: A language and environment for statistical computing 2008 Vienna, Austria: R Foundation for Statistical Computing

[B37] Shifren JL Rifai N Desindes S McIlwain M Doros G Mazer NA A comparison of the short-term effects of oral conjugated equine estrogens versus transdermal estradiol on C-reactive protein, other serum markers of inflammation, and other hepatic proteins in naturally menopausal women J Clin Endocrinol Metab 2008 93 5 1702 1710 10.1210/jc.2007-2193 18303079

[B38] Walsh BW Schiff I Rosner B Greenberg L Ravnikar V Sacks FM Effects of postmenopausal estrogen replacement on the concentrations and metabolism of plasma lipoproteins N Engl J Med 1991 325 17 1196 1204 1922206 10.1056/NEJM199110243251702

[B39] Fotherby K Bioavailability of orally administered sex steroids used in oral contraception and hormone replacement therapy Contraception 1996 54 2 59 69 10.1016/0010-7824(96)00136-9 8842581

[B40] Thierry-Mieg D Thierry-Mieg J AceView: a comprehensive cDNA-supported gene and transcripts annotation Genome Biol 2006 7 Suppl 1 S12 11-14 10.1186/gb-2006-7-s1-s12 16925834 PMC1810549

[B41] Zhang X Merkler KA McLean MP Characterization of regulatory intronic and exonic sequences involved in alternative splicing of scavenger receptor class B gene Biochem Biophys Res Commun 2008 372 1 173 178 10.1016/j.bbrc.2008.05.007 18477479

[B42] Carroll MD Lacher DA Sorlie PD Cleeman JI Gordon DJ Wolz M Grundy SM Johnson CL Trends in serum lipids and lipoproteins of adults, 1960-2002 JAMA 2005 294 14 1773 1781 10.1001/jama.294.14.1773 16219880

[B43] Grove J Huby T Stamataki Z Vanwolleghem T Meuleman P Farquhar M Schwarz A Moreau M Owen JS Leroux-Roels G Scavenger receptor BI and BII expression levels modulate hepatitis C virus infectivity J Virol 2007 81 7 3162 3169 10.1128/JVI.02356-06 17215280 PMC1866051

[B44] Codes L Matos L Parana R Chronic hepatitis C and fibrosis: evidences for possible estrogen benefits Braz J Infect Dis 2007 11 3 371 374 10.1590/S1413-86702007000300014 17684642

[B45] Hankinson SE Manson JE Spiegelman D Willett WC Longcope C Speizer FE Reproducibility of plasma hormone levels in postmenopausal women over a 2-3-year period Cancer Epidemiol Biomarkers Prev 1995 4 6 649 654 8547832

[B46] Muti P Trevisan M Micheli A Krogh V Bolelli G Sciajno R Berrino F Reliability of serum hormones in premenopausal and postmenopausal women over a one-year period Cancer Epidemiol Biomarkers Prev 1996 5 11 917 922 8922301

[B47] Mortola JF Laughlin GA Yen SS A circadian rhythm of serum follicle-stimulating hormone in women J Clin Endocrinol Metab 1992 75 3 861 864 10.1210/jc.75.3.861 1517378

