# Supplementary figures and images for: Impact of gene variants on sex-specific regulation of human Scavenger receptor class B type 1 (SR-BI) expression in liver and association with lipid levels in a population-based study
Source: BMC Med Genet. 2010 Jan 19;11:9. doi: 10.1186/1471-2350-11-9 (PMC2822818; doi:10.1186/1471-2350-11-9)

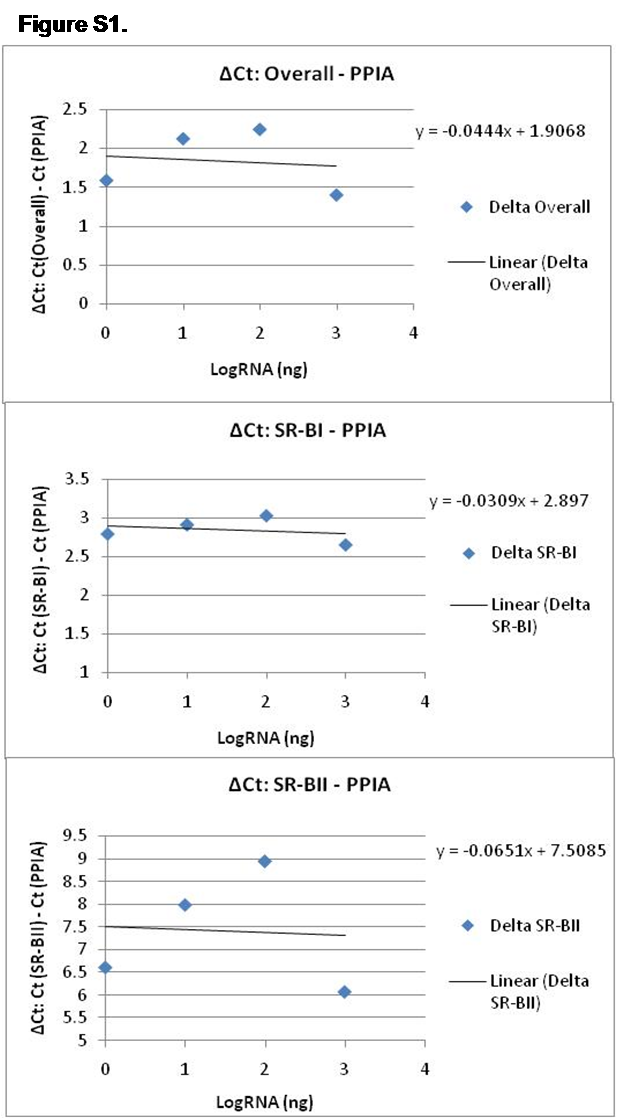

Supplement: Additional file 1 — Figure S1. Validation curves of the ΔΔ real-time gene expression assays. Shown are curves for overall SCARB1, SR-BI and SR-BII versus endogenous control (PPIA). Relative efficiency plots were formed by plotting the log input amount (ng of total RNA) versus the ΔCt (Ct SCARB1 - Ct PPIA), for example. The slopes are <0.1 which indicates the validation of the ΔΔCt calculation relative to the reference controls in the range between 1-100 ng RNA. [file 1471-2350-11-9-S1.TIFF]
